# The Association of Women’s Empowerment with Stillbirths in Nepal

**DOI:** 10.1007/s10995-019-02827-z

**Published:** 2019-11-29

**Authors:** Abhishek Gurung, Kiran Bajracharya, Rejina Gurung, Shyam Sundar Budhathoki, Naresh Pratap KC, Parashu Ram Shrestha, Ashish KC

**Affiliations:** 1 Golden Community, Lalitpur, Nepal; 2 Midwifery Society of Nepal, Kathmandu, Nepal; 3 School of Public Health and Community Medicine, B.P Koirala Institute of Health Sciences, Dharan, Nepal; 4 Society of Public Health Physicians Nepal, Kathmandu, Nepal; 5 Ministry of Health and Population, Government of Nepal, Kathmandu, Nepal; 6 Department of Women’s and Children’s Health, International Maternal and Child Health, University Hospital, 751 85 Uppsala, Sweden

**Keywords:** Women’s empowerment, Women’s autonomy, Stillbirths, Nepal

## Abstract

**Introduction:**

Globally, 2.6 million stillbirths occur each year. Empowering women can improve their overall reproductive health and help reduce stillbirths. Women empowerment has been defined as women’s ability to make choices in economic decision-making, household and health care decision-making. In this paper, we aimed to evaluate if women’s empowerment is associated with stillbirths.

**Methods:**

Data from 2016 Nepal Demographic Health Surveys (NDHS) were analysed to evaluate the association between women’s empowerment and stillbirths. Equiplots were generated to assess the distribution of stillbirths by wealth quintile, place of residence and level of maternal education using data from NHDS 1996, 2001, 2006, 2011 and 2016 data. For the association of women empowerment factors and stillbirths, univariate and multivariate analyses were conducted.

**Results:**

A total of 88 stillbirths were reported during the survey. Univariate analysis showed age of mother, education of mother, age of husband, wealth index, head of household, decision on healthcare and decision on household purchases had significant association with stillbirths (p < 0.05). In multivariate analysis, only maternal age 35 years and above was significant (aOR 2.42; 1.22–4.80). Education of mother (aOR 1.48; 0.94–2.33), age of husband (aOR 1.54; 0.86–2.76), household head (aOR 1.51; 0.88–2.59), poor wealth index (aOR 1.62; 0.98–2.68), middle wealth index (aOR 1.37; 0.76–2.47), decision making for healthcare (aOR 1.36; 0.84–2.21) and household purchases (aOR 1.01; 0.61–1.66) had no any significant association with stillbirths.

**Conclusions:**

There are various factors linked with stillbirths. It is important to track stillbirths to improve health outcomes of mothers and newborn. Further studies are necessary to analyse women empowerment factors to understand the linkages between empowerment and stillbirths.

## Significance

This paper used data from the Nepal Demographic and Health Survey 2016 to assess the association between women’s empowerment and stillbirths in Nepal. We reported no any significant association for empowerment factors with stillbirth. However, it will be important to conduct large-scale surveys to determine the associations between women empowerment factors and stillbirths.

## Introduction

Every year, around 2.6 million stillbirths occur worldwide, with 98% occurring primarily in low and middle-income countries (LMICs) (Blencowe et al. 2016). World Health Organization (WHO) defines stillbirths as no signs of life in babies at or after 28 weeks of gestation (World Health Organization 2014). Most stillbirths in LMICs are intrapartum and cause profound damage and grief (Roberts et al. 2012). In South Asia, 59% of stillbirths occur in the intrapartum period (Lawn et al. 2016).

Stillbirths cause many women significant distress, potentially resulting in mental health issues (Roberts et al. 2012). In some societies, having a stillbirth can lead to abuse and even abandonment by husbands (Kiguli et al. 2016; Roberts et al. 2012). Poorer women are already at a disadvantage as they suffer more stillbirths than women who are from well-off backgrounds (Flenady et al. 2011). This is probably due, at least in part, to less access to prenatal care (KC et al. 2016). Additional risk factors for stillbirths include previous stillbirths (Aminu et al. 2014) and advanced maternal age, specifically being above 35 years of age (Flenady et al. 2011).

Women who are economically active play a more direct role in household decision-making and therefore have better bargaining power in terms of education and access to health care (Mainuddin et al. 2015). Mother’s level of education also plays an important role in health care utilisation (Chakraborty et al. 2003). While several studies have looked at women’s empowerment and pregnancy-related outcomes, none have looked at the association between empowerment and stillbirths as a primary outcome. We aimed at evaluating the association between women’s empowerment, socioeconomic status and stillbirths in Nepal to provide an overview of possible links between empowerment factors and stillbirths.

## Methods

The study is based on data from the 2016 Nepal Demographic and Health Survey (NDHS) (Ministry of Health 2017).

### Data Collection

The NDHS is a cross-sectional survey conducted every 5 years in Nepal and many other countries. The 2016 survey used simple stratified sampling with two stages in rural areas and three stages in urban areas yielding 14 sampling strata. A total of 12,862 women aged 15–49 were interviewed during the survey. Among them, 5086 pregnancies were beyond 7 months’ gestation. The response rate to interview was 98%.

### Data Management

The primary dataset was downloaded from the DHS website after providing a summary of the research project to MEASURE DHS. All indices linked to empowerment were selected for further analysis. Indices of women empowerment were based on three broad dimensions (1) economic Decision-making to purchase household items, (2) decision-making for seeking health care and (3) decision-making on physical mobility to visit relatives (Hameed et al. 2014). The variables extracted from the dataset were: maternal age, maternal level of education (with uneducated referring no formal education), husband’s age, husband’s occupation, wealth (defined by household asset score categorized by centile), sex of household head, place of residence (urban or rural), ecological zone and women’s autonomy (defined by decision making ability related to health care, household purchases and visiting relatives).

### Data Analysis

The datasets were weighted before performing analysis. Similarly, the sample domain and cluster design were also addressed creating a complex sample analysis (CSA) plan before performing the analysis. The socio-demographic and empowerment characteristics were analysed for stillbirths using binary logistic regression. Only the indicators that could have a considerable impact on women’s positions in their families, and that could have a direct or indirect impact on pregnancy outcomes were chosen. The association was considered significant for p-value < 0.05. Any missing values were excluded from analysis. Multiple regression analysis was done for the variables that were significant in univariate analyses. All analyses were carried out in SPSS version 23.

Equity data analysis was also carried out using ‘equiplots’ to analyse inequalities between different socioeconomic groups, geographical strata and education levels based on data from the NDHS 1996, 2001, 2006, 2011 and 2016. This allowed for the presentation of equality gaps between different strata.

## Results

**Fig. 1:**
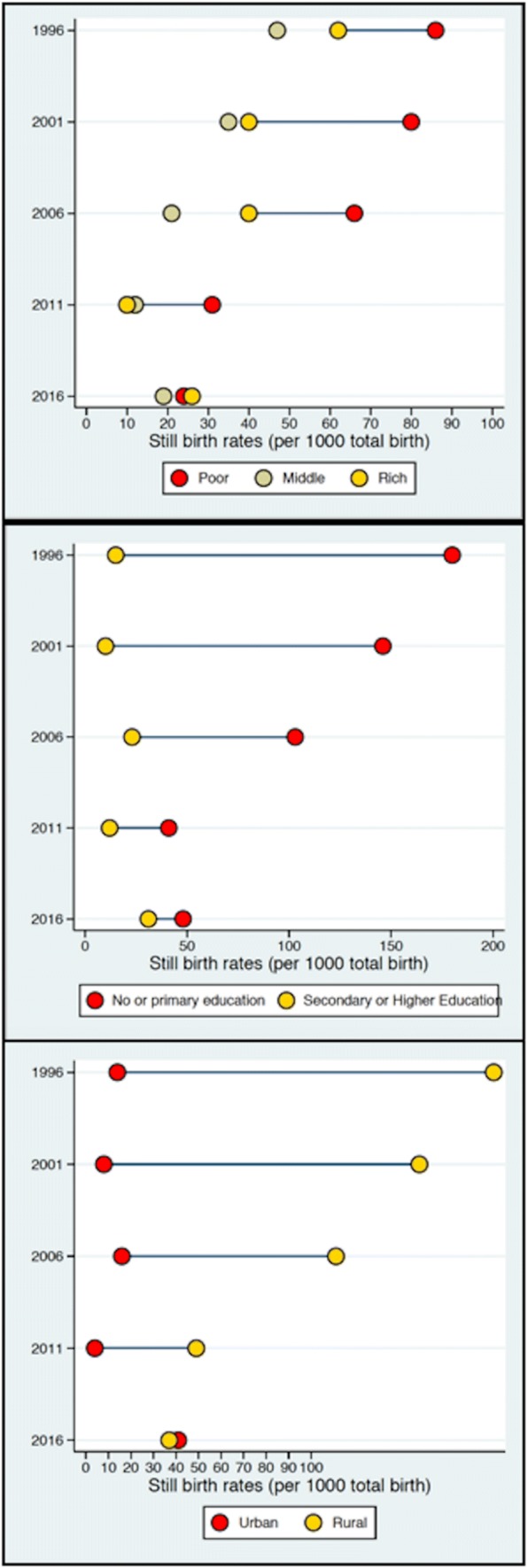
Trends in stillbirths in Nepal (in serial order), by wealth index, education level and place of residence per 1000 live births (1996–2016 NDHSs)

A total of 12, 862 women were interviewed during the NDHS survey. Among them, 88 stillbirths were reported. In univariate analysis, socio-demographic factors such as age of mother, education of mother, age of husband, wealth index, all showed significant association with stillbirths (p < 0.05). Similarly, empowerment factors such as head of household, decision on healthcare and decision on household purchases also showed significant association with stillbirths (p < 0.05) (Table 1). The women with reported higher rates of stillbirths were from urban areas and the Terai (plains) region and having less education regardless of wealth status. Disparities in stillbirth rates were found between women by level of education, wealth index and place of residence though decreasing over the years. The equiplots were generated based on the data from NDHS 1996, 2001, 2006, 2011 and 2016 (Fig. 1).

**Table 1:** Socio-demographic and empowerment characteristics

Variables	Stillbirth (n = 88)	No stillbirth (n = 12774)	Total (n = 12862)	p-value	OR (95% CI)
Age of mother					
< 35 years	72 (81.8%)	8718 (68.2%)	8790 (68.3%)		Ref
≥ 35 years	16 (18.2%)	4056 (31.8%)	4072 (31.7%)	0.01	0.28–0.82
Education of mother					
Educated	49 (55.7%)	8532 (66.8%)	8581 (66.7%)		Ref
Uneducated	39 (44.3%)	4242 (33.2%)	4281 (33.3%)	0.04	(1.03–2.40)
Age of husband					
≥ 40 years	23 (26.1%)	7504 (58.7%)	7527 (58.5%)		Ref
< 40 years	65 (73.9%)	5270 (41.3%)	5335 (41.5%)	< 0.001	2.47–6.37
Education of husband (n = 9852)					
Educated	67 (77.0%)	8210 (84.1%)	8227 (84.0%)		Ref
Uneducated	20 (23.30%)	1555 (15.9%)	1575 (16.0%)	0.07	(0.97–2.63)
Employment of husband (n = 8003)					
Employed	65 (95.6%)	7574 (95.5%)	7639 (95.5%)		Ref
Unemployed	3 (4.4%)	361 (4.5%)	364 (4.5%)	0.83	0.26–2.95
Wealth index					
Rich	26 (29.5%)	5540 (43.4%)	5566 (43.3%)		Ref
Poor	41 (46.6%)	4660 (36.5%)	4701 (36.5%)	0.01	1.16–3.09
Middle	21 (23.9%)	2574 (20.2%)	2595 (20.2%)	0.07	0.96–3.05
Head of household					
Female	18 (20.5%)	3978 (31.1%)	3996 (31.1%)		Ref
Male	70 (79.5%)	8796 (68.9%)	8866 (68.9%)	0.04	1.03–2.87
Ecological zone					
Mountain	3 (3.4%)	771 (6.0%)	774 (6.0%)		Ref
Hill	32 (0.6%)	5524 (43.3%)	5556 (43.2%)	0.63	0.43–4.07
Terai	53 (60.2%)	6479 (50.7%)	6532 (50.8%)	0.27	0.62–5.57
Place of residence					
Urban	47 (53.4%)	8025 (62.8%)	8072 (62.8%)		Ref
Rural	41 (46.6%)	4749 (37.2%)	4790 (37.2%)	0.08	0.96–2.21
Decision on healthcare (n = 9874)					
Husband and wife together	39 (44.3%)	5663 (57.9%)	5702 (57.7%)		Ref
Husband alone	49 (55.7%)	4123 (42.1%)	4172 (42.3%)	0.01	1.14–2.65
Decision on household purchases (n = 9875)					
Husband and wife together	36 (40.9%)	5195 (53.1%)	5231 (53.0%)		Ref
Husband alone	52 (59.1%)	4592 (46.9%)	4644 (47.0%)	0.03	1.05–2.47
Decision on visiting family/relatives (n = 9875)					
Husband and wife together	46 (52.3%)	5446 (55.6%)	5492 (55.6%)		Ref
Husband alone	42 (47.7%)	4341 (44.4%)	4383 (44.4%)	0.47	0.77–1.78
Physically forced for unwanted sex (n = 3801)					
No	26 (86.7%)	3512 (93.1%)	3538 (93.1%)		Ref
Yes	4 (13.3%)	259 (6.9%)	263 (6.9%)	0.20	0.69–5.88

The variables that were significant in the univariate analyses were used in the multivariate analysis. Only age of mother was significant in the multivariate analysis. In mothers aged 35 years and above, the risk of stillbirths was 2.42 times (aOR 2.42; 1.22–4.80) in comparison to mothers aged less than 35 years. Multivariate analysis showed socio-demographic factors such as education of mother (aOR 1.48; 0.94–2.33), age of husband (aOR 1.54; 0.86–2.76), household head (aOR 1.51; 0.88–2.59), poor wealth index (aOR 1.62; 0.98–2.68) and middle wealth index (aOR 1.37; 0.76–2.47) had no any significant association with stillbirths. Further, empowerment factors such as decision making for healthcare (aOR 1.36; 0.84–2.21) and household purchases (aOR 1.01; 0.61–1.66) had no significant association with stillbirths (Table 2).

**Table 2:** Multivariate analysis of empowerment factors associated with stillbirth (n = 9904)

Variables	β—coefficient	p-value	AOR (95% CI)
Age of mother			
< 35 years	Ref		
≥ 35 years	0.884	0.011	2.42 (1.22–4.80)
Education of mother			
Educated	Ref		
Uneducated	0.393	0.090	1.48 (0.94–2.33)
Age of husband			
≥ 40 years	Ref		
< 40 years	0.430	0.149	1.54 (0.86–2.76)
Household head			
Female	Ref		
Male	0.412	0.135	1.51 (0.88–2.59)
Wealth index			
Rich	Ref	0.174	
Poor	0.482	0.062	1.62 (0.98–2.68)
Middle	0.314	0.296	1.37 (0.76–2.47)
Decision for healthcare			
Husband and wife together	Ref		
Husband alone	0.309	0.212	1.36 (0.84–2.21)
Decision for household purchases			
Husband and wife together	Ref		
Husband alone	0.006	0.983	1.01 (0.61–1.66)

## Discussion

The study describes the socio-demographic and empowerment factors associated with stillbirths based on the NDHS 2016 data. Disparities in stillbirth rates were found between women by level of education, wealth index and place of residence. However, better access to education, improving socioeconomic status and more people living in urban areas could be the reason why the disparity is decreasing over the years as reported by the NDHS report (Ministry of Health 2017).

The study analysed empowerment related factors for stillbirths. Findings showed that the risk of stillbirth was significant for mothers aged 35 years and above. Waldenström et al. based on a population-based registry in Sweden, have found that advanced maternal age is a risk factor for stillbirth, especially for first time mothers (Waldenström et al. 2015). It has also been corroborated by a meta-analysis which mentioned that women aged 35 years and more contributed to stillbirth (Flenady et al. 2011). Similar findings have been reported from other studies (Aminu et al. 2014; KC et al. 2016; Lawn et al. 2016; Yudkin et al. 1987).

Education has an important role to play in determining a woman’s status in their families and society and for improving communication between husbands and wives (Furuta and Salway 2006). However, educational status of mother had no significant association with stillbirths in our study because there are many other factors impacting fertility outcomes (Shimamoto and Gipson 2015). Education alone is not enough for a woman to determine her fertility choices (Woldemicael 2009). However, a study conducted in a tertiary hospital by KC et al. found that women with < 5 years of education had a significant association with antepartum stillbirths (KC et al. 2015). A systematic literature reviews also showed similar findings (Aminu et al. 2014).

Women from any wealth group or women being the household head had no any association with stillbirths in our study. A Canadian study also found no linkages between socioeconomic status and adverse pregnancy outcomes citing minimal impact (Campbell et al. 2018). However, another cohort study conducted in Australia showed that low socioeconomic status was with stillbirths (Davies-Tuck et al. 2017). A Zambian verbal autopsy study also corroborated the finding though they included mortalities for all children under the age of two (Turnbull et al. 2011). Heading the household can positively impact women’s health, including stillbirth prevention, though they recommend that further studies are necessary to understand the associations (De Bernis et al. 2016). Women are generally gaining more autonomy, and autonomy is an important predictor of reproductive health status in developing countries like Nepal (Rahman 2012). A recent study conducted in Ethiopia found that women from a wealthy background were less likely to have stillbirths (Lakew et al. 2017). Other studies have also found similar linkages for wealth status and stillbirths (Aminu et al. 2014; KC et al. 2016; Kwagala et al. 2016).

Women with better decision making for healthcare and household purchases had no any significant association with stillbirths in our study. A study conducted in Nigeria found that empowered women had more possibility of delivering in a health facility and seeking safer birth practices, however numbers varied across country (Corroon et al. 2014). Further, joint decision-making during pregnancy and childbirth means better reproductive health outcomes for women (Story and Burgard 2012). A study by Fotso et al. demonstrated similar findings (Fotso et al. 2009). Furthermore, women’s fertility choices may be limited if their husbands and mothers-in-law (Woldemicael 2009) control or disapprove of their actions, irrespective of a woman’s educational status. A Bangladesh study concluded that there are negative aspects related to seeking antenatal care and health services if the decision is made by the husband only (Story and Burgard 2012). Thus, efforts should focus on involving male partner more in seeking and obtaining maternal health services.

This study has several limitations. It is based on the analysis of secondary data—the 2016 NDHS. The NDHS is an interviewer-administered survey, which can result in social interest bias, with interviewees being reluctant to reveal sensitive information like intimate partner violence and other pregnancy outcomes (Zakar et al. 2015). Also, the NDHS women’s questionnaires only had a single category and did not categorize stillbirths into antepartum and intrapartum stillbirths, so the association of women’s empowerment with different types of stillbirths cannot be analysed. There might also have been bias in the reporting of stillbirths due to the retrospective nature of the interviews. Also, there could have been recall bias leading to fewer reported cases from mothers; and misclassification bias due to interviewers diagnosing deaths based on mothers’ reports.

The DHS stillbirth figures are based on retrospective pregnancy histories over the previous 5 years and may be inaccurate. Further, there is very little research on stillbirth and policy level implications are also scarce (McClure et al. 2009). It is also important to consider verbal autopsies with mothers who have stillbirths to get a better perspective on the causes. Even with DHS being conducted in many countries, no verbal autopsies were done in the last 5 years irrespective of the recommendations (Lawn et al. 2010, 2011). Having said that, DHS data are the largest source of national level data from LMICs (Lawn et al. 2010) with very little availability of national level estimates (Lawn et al. 2011), this will add to the literature.

Under the Every Newborn Action Plan (ENAP), Nepal aims to reduce stillbirths to 12 or less per 1000 births by 2030. However, the focus so far has been largely on reducing newborn deaths rather than stillbirths. Our findings showed no any significant associations for women empowerment factors related to stillbirths. Having said that, the need is to include better counting measures for tracking stillbirths (Stanton et al. 2006). Since, stillbirths are related to maternal and newborn mortalities, it will be crucial to reduce the numbers for better survival of mothers and newborn (McClure et al. 2007). Large scale studies aimed at understanding the linkages between empowerment and stillbirths are necessary.

## Abbreviation

ANC: Antenatal care
CSA: Complex sample analysis
ENAP: Every newborn action plan
LMIC: Low and middle-income countries
NDHS: Nepal demographic and health survey
aOR: Adjusted odds ratio
